# Identification of the SUMO E3 ligase PIAS1 as a potential survival biomarker in breast cancer

**DOI:** 10.1371/journal.pone.0177639

**Published:** 2017-05-11

**Authors:** Ayan Chanda, Angela Chan, Lili Deng, Elizabeth N. Kornaga, Emeka K. Enwere, Donald G. Morris, Shirin Bonni

**Affiliations:** 1 Arnie Charbonneau Cancer Institute and Biochemistry and Molecular Biology, University of Calgary, Calgary, Alberta, Canada; 2 Translational Laboratories, Tom Baker Cancer Centre, Alberta Health Services, Calgary, Alberta, Canada; 3 Department of Oncology, Alberta Health Services, Calgary, Alberta, Canada; Florida International University, UNITED STATES

## Abstract

Metastasis is the ultimate cause of breast cancer related mortality. Epithelial-mesenchymal transition (EMT) is thought to play a crucial role in the metastatic potential of breast cancer. Growing evidence has implicated the SUMO E3 ligase PIAS1 in the regulation of EMT in mammary epithelial cells and breast cancer metastasis. However, the relevance of PIAS1 in human cancer and mechanisms by which PIAS1 might regulate breast cancer metastasis remain to be elucidated. Using tissue-microarray analysis (TMA), we report that the protein abundance and subcellular localization of PIAS1 correlate with disease specific overall survival of a cohort of breast cancer patients. In mechanistic studies, we find that PIAS1 acts via sumoylation of the transcriptional regulator SnoN to suppress invasive growth of MDA-MB-231 human breast cancer cell-derived organoids. Our studies thus identify the SUMO E3 ligase PIAS1 as a prognostic biomarker in breast cancer, and suggest a potential role for the PIAS1-SnoN sumoylation pathway in controlling breast cancer metastasis.

## Introduction

Metastasis is the major cause of cancer related mortality [1]. Despite intensive scrutiny, the mechanisms that control the invasive growth and metastatic potential of breast cancer remain incompletely understood [2]. Epithelial-mesenchymal transition (EMT) is thought to play a key role in tumor metastasis [3–5]. EMT promotes the transdifferentiation of epithelial cells into migratory, invasive, and mesenchymal-like cells [3]. Carcinoma cells undergoing EMT can escape from primary tumor sites, enter the circulation, and then move out to invade distant sites where secondary tumors or metastases form [6,7]. Thus, identifying regulators of EMT should provide insights into the mechanisms that control tumor metastasis and hence patient survival.

Transforming Growth Factor beta (TGFβ) is a versatile cytokine that has a biphasic role in cancer [8]. TGFβ induces cell cycle arrest in diverse cell types including epithelial cells, which contributes to TGFβ's tumor suppressive role [9]. On the other hand, TGFβ can promote cancer cell invasion and metastasis, especially at the later stages of cancer [8,9], via induction of EMT.

The small ubiquitin like modifier (SUMO) pathway has emerged as a key regulator of TGFβ-induced EMT in non-transformed epithelial cells and potentially in tumor cells [10–12].

The protein inhibitor of activated stats (signal transducers and activator of transcription) or PIAS represents a well-studied family of SUMO E3 ligases [13,14]. In particular, the PIAS family member PIAS1 associates with and promotes the sumoylation of the transcriptional coregulator SnoN (Ski-related novel protein N), a key component of TGFβ signalling and responses [15,16]. Importantly, PIAS1 acts via sumoylation of SnoN to suppress TGFβ-induced EMT of non-transformed epithelial cells [12]. Recent evidence suggests that PIAS1 suppresses the invasive and metastatic growth of human breast cancer cells in three-dimensional-derived multicellular structures and xenograft animal model, respectively [11]. These studies have raised the important questions of the value of PIAS1 as a prognostic/therapeutic biomarker in breast cancer, and the mechanisms by which PIAS1 suppresses the invasiveness and metastasis of breast cancer cells.

In this study, we identify PIAS1 as a biomarker that predicts disease-specific overall survival (DSOS) in endocrine-treated breast cancer patients. In mechanistic studies, we find that PIAS1 acts via sumoylation of SnoN to suppress the invasive growth of human breast cancer cell-derived organoids. Collectively, our findings suggest the PIAS1-SnoN sumoylation pathway may play a fundamental role in suppression of human breast cancer invasiveness and potentially metastasis, and identify PIAS1 as a biomarker that predicts improved survival of breast cancer patients.

## Materials and methods

### Plasmids

CMV-based plasmids to express FLAG-tagged wild type SnoN (WT), SUMO loss of function SnoN, in which Lysines 50 and 383 are converted to arginine residues (KdR), wild type PIAS1 (WT), and SUMO E3 ligase mutant PIAS1, in which Cysteine 350 is converted to serine (CS), and U6-based plasmids, with enhanced green fluorescent protein (GFP), to express short hairpin RNA (shRNA) against SnoN or PIAS1 have been described previously [11,17–19]. To establish SnoN-expressing stable MDA-MB-231 cells, a pCaGip vector containing a cDNA to express puromycin resistance marker was employed to generate constructs containing cDNA encoding SnoN (WT), a SUMO loss-of-function SnoN (KdR), or a SUMO gain-of-function stable fusion SUMO-SnoN protein. The puromycin resistance marker and protein of interest are encoded by a bicistronic transcript containing Internal Ribosomal Entry Site (IRES) as part of the pCaGip vector [11,12]. Generation of MDA-MB-231 cells stably expressing PIAS1 (WT) or PIAS1 (CS) have been described [11].

### Cell lines and transfections

Human embryonic kidney epithelial 293T cells were cultured in Dulbecco’s modified Eagle’s medium with high glucose and L-glutamine (DMEM) (Invitrogen, Canada) supplemented with 10% fetal bovine serum (FBS, Invitrogen). MDA-MB-231 human breast cancer cells, purchased from American Type Culture Collection (ATCC, Manassas, VA, USA), were maintained in 10% FBS-supplemented DMEM. 293T cells were transiently transfected using the calcium phosphate precipitation method, and MDA-MB-231 cells were transfected using Lipofectamine LTX Plus reagents (Invitrogen, Canada) [12,16]. MDA-MB-231 cells were transfected with pCaGip vector or pCaGip plasmid encoding SnoN (WT), SnoN (KdR) or SUMO-SnoN using Lipofectamine LTX Plus reagents and incubated with1 μg/ml puromycin (Invitrogen, Canada) containing complete medium [11].

### Patient cohort

The Calgary tamoxifen breast cancer cohort is a large retrospective cohort containing patients diagnosed between 1985 and 2000, and has been described in detail previously [20]. This study was reviewed and approved by the Health Research Ethics Board of Alberta. The Calgary 65/65 cohort is a subset of 130 patients, in which 65 patients had no recurrence and 65 patients had a recurrence at 5 years [21]. Only patients with confirmed invasive breast cancer with surgical intervention, and who received adjuvant tamoxifen therapy (20mg patient orally (p.o.)/day) were included. For the 130 patients who met these criteria, a single TMA was constructed using replicate 0.6-mm cores taken from their archival tissue blocks [22]. Patients from this 130 cohort were excluded from analysis if they had any prior cancer diagnosis (except non-melanoma skin cancer), or if they received neo- or adjuvant-chemotherapy, to obtain a uniform tamoxifen-only primary breast cancer cohort for analysis. A total of 108 patients met inclusion/exclusion criteria.

### Indirect immunofluorescence

96-well plate-seeded MDA-MB-231 cells were subjected to indirect immunofluorescence as described [12]. Briefly, MDA-MB-231 cells were fixed with 4% formaldehyde (Thermo Fisher, Canada), permeabilized with 0.2% Triton-X100, and blocked using 5% bovine serum albumin (BSA, Sigma) and 5% calf serum (VWR, Canada) in phosphate buffered saline (PBS) [12]. Subcellular localization and abundance of PIAS1 were determined by incubating MDA-MB-231 cells with a rabbit anti-PIAS1 antibody (Abcam, USA) followed by Cy3-conjugated anti-rabbit IgG (Millipore) using a well-established indirect immunofluorescence protocol [12]. Cells were incubated with the DNA fluorescent dye Hoechst 33342 (Invitrogen) to visualize their nuclei. GFP expression was used as a readout of transfection efficiency. Immunofluorescent images of cells were captured using a fluorescence microscope with a 40 X objective lens (Zeiss Axiovert 200M). For each experiment, each fluorophore exposure time was kept constant.

### Reference TMA establishment

Equal concentrations of hemocytometer-counted trypsinized-suspension of MDA-MB-231 cells, expressing increasing amounts of PIAS1, were subjected to two PBS washes to remove excess trypsin involving two rounds of 5min X 1200 RPM centrifugation at 4°C and resuspension of pellet in ice-cooled PBS. Washed cell pellets were then resuspended and fixed in 10% formalin (Thermo Fisher Scientific, Burlington, ON, Canada) by incubating for 60 minute on ice, followed by two rounds of room-temperature PBS wash as described above. Cells were counted again during the last PBS wash using hemocytometer. Fixed, washed and dried cell pellets were homogenously resuspended in 65°C molten Histogel (Thermo Fisher, Canada) at approximately 1x10^7^ cells/100 μL of Histogel. The gel-embedded cells were allowed to set at 4°C after which they were suspended in 70% ethanol and kept at 4°C until processing using paraffin-embedded breast cancer tissue samples procedure (see below).

### Fluorescence immunohistochemistry

After tissue microarray construction, 4 μm thick sections were cut from the TMA block and deparaffinized in xylene, rinsed in ethanol, and rehydrated. Heat-induced epitope retrieval was performed by heating slides to 121°C in a citrate-based buffer (pH6) Target Retrieval Solution (Dako, Mississauga, ON, Canada) for 3 minutes in a decloaking chamber (Biocare Medical, Concord, CA, USA). Slides were stained using a Dako Autostainer Link 48. Endogenous peroxidase activity was quenched with a 10 minute incubation of peroxidase block (a component of the DAKI Envision™+ System) followed by a 15 minute protein block (Signal Stain, Cell Signaling, Danvers, MA, USA) to eliminate non-specific antibody binding. All incubations were performed at room temperature. Slides were washed with wash buffer (Tris-buffered saline containing 0.05% Tween-20 (TBST, DAKO)) and then incubated for 30 minutes with Signal Stain protein block (Cell Signaling) containing a 1:2000 dilution of Rabbit anti-PIAS1 monoclonal antibody, clone EPR2580 (2) (Abcam, Cambridge, MA, USA). After three washes, a goat anti-rabbit horseradish peroxidase (HRP)-conjugated secondary antibody from the DAKO EnVision™+ System was applied for 30 minutes. Slides were again washed in TBST and treated for 5 minutes with TSA-Plus Cy5 tyramide signal amplification reagent (1:100, PerkinElmer, Woodbridge, ON, Canada). To identify tumor cells, slides were further incubated for 30 minutes with the pan-cytokeratin antibody, followed by three washes and a 30-minute incubation of a 1:200 dilution of Alexa-555 conjugated goat anti-mouse antibody (Life Technologies, Burlington, ON, Canada). After three washes in TBST, the TMA slides were mounted with ProLong™ Gold anti-fade mounting medium containing diamidino-2-phenylindole (DAPI) (Life Technologies) and stored at 4°C overnight to set before scanning.

### Automated image acquisition and analysis

Automated image acquisition was performed using an Aperio Scanscope FL (Aperio Inc., Vista, CA, USA). Seamless high-resolution slide images were acquired using the Scanscope FL 8/10-bit monochrome TDI line-image capture camera using filters specific for DAPI to define the nuclear compartment, Cy3 to define cytokeratin in the tumor cytosolic compartment, and Cy5 to define the target antibody PIAS1. Images were then analysed using the AQUAnalysis® program, version 2.3.4.1 as previously described (PMID: 12389040) and Indica Labs HALO® program version 2.0.1032. Briefly, a tumor-specific mask was generated to distinguish the breast cancer cells from surrounding stromal tissue by thresholding the pan-cytokeratin images. Thresholding created a binary mask that identified the presence or absence of tumor cells by the presence of a pixel that was ‘on’ or ‘off’, respectively. Thresholding levels were verified and adjusted, if necessary, by spot-checking a small sample of images to determine an optimal threshold value. All images were then processed using this optimal threshold value and all subsequent image manipulations involved only image information from the masked area. Images were validated according to the following: 1) >10% of the tissue area is pan-cytokeratin positive, 2) >50% of the image was usable (i.e. not compromised due to overlapping or out of focus tissue). Unusable areas within each image were manually cropped so that they were excluded from the final analysis. After review and image analysis validation, there were 96 patients that had usable results for subsequent statistical analysis.

### Sumoylation, immunoprecipitation and immunoblotting assays

For *in vivo* sumoylation assays, MDA-MB-231 cells were treated without or with 100pM TGFβ1 (R&D Systems, USA), TGFβ receptor I kinase inhibitor SB431542 (KI) (Sigma, Canada), alone or together for 12 hours. Subsequently, the cells were lysed in TNTE (50 mM Tris, 150 mM NaCl, and 1 mM EDTA) buffer containing 0.5% Triton X-100 along with protease and phosphatase inhibitors, and 0.1% SDS [10–12,18,19]. In addition, 20 mM of the isopeptidase inhibitor n-ethylmaleimide (NEM) (Calbiochem, Canada) was included in the lysis buffer to inhibit the SENPs and preserve sumoylation of substrates.[10,12,18]. Cell extracts were collected in Eppendorf tubes and centrifuged at 14000 × g for 10 min at 4°C. Supernatants of cell lysates were collected and subjected to rabbit anti-SnoN (Santa Cruz Biotechnology, Dallas, TX, USA, H-317) immunoprecipitation at 4°C, after saving 10% of the supernatant for protein quantification and Bradford protein expression analysis using dye reagent (Bio-Rad Laboratories, Canada) and a BSA standard curve [10,12,18,23]. In experiments that did not assess SnoN sumoylation, immunoblotting analysis was carried out on cell lysates prepared as above but in the absence of NEM. Equivalent protein amounts of lysates or immunoprecipitation complexes were resolved by SDS-PAGE followed by transfer to nitrocellulose (NC) membranes. Specific proteins on NC membranes were subjected to mouse anti-FLAG (Sigma, Canada), rabbit anti-PIAS1 (Abcam, USA), rabbit anti-SnoN (Santa Cruz, Canada), mouse anti-SUMO1 (Developmental Studies Hybridoma Bank, IO, USA) or mouse anti-actin (Santa-Cruz), as the primary antibody, and HRP-conjugated goat anti-mouse or donkey anti-rabbit IgG (Jackson Laboratories) as secondary antibodies, followed by incubation in Enhanced Chemiluminescence (ECL) (Millipore) reagent and light signal detection using a VersaDoc 5000 Imager (Bio-Rad Laboratories). Densitometry was performed using Quantity One software (Bio-Rad Laboratories) [10–12,18,24].

### Three-dimensional cultures

Three-dimensional cultures of MDA-MB-231 cells were prepared by first coating 96-well flat-bottom, ultra-low attachment plates (BD Biosciences, ON, Canada) with 50 μl of 30% growth-factor-reduced Matrigel (3 mg/ml) (BD Bioscience) diluted in complete medium (DMEM containing 10% FBS, penicillin, streptomycin and amphotericin B) (Invitrogen) and incubating for 1 h in a 5% CO_2_ humidified incubator at 37°C to form a 1 mm thick 30% Matrigel bed. The 30%-diluted Matrigel containing medium was kept on ice prior to transfer to wells of the 96-well tissue culture plate. 50 μl of 50% Matrigel in complete medium-containing approximately 300 MDA-MB-231 cells were carefully layered on top of the Matrigel bed in the 96-well plate. 50 μl of complete medium was layered on top 1 h later. The following day and at every third day, three-dimensional cultures received 50 μl fresh complete medium alone, or with 100 pM TGFβ. After an overall estimate of the total number (approximately 50) and assessment of deformed versus non-deformed growth phenotypes of the 8-day live three-dimensional multicellular structures in each well using light microscopy at 30 X objective (Olympus IX70), differential interference contrast (DIC) images were captured of 8 representative organoids. For each experimental condition, organoids showing any protrusions and invasiveness were denoted as deformed organoids, thus indicating invasive behaviour, and those with smooth surface and no budding or protrusion were denoted as non-deformed organoids, i.e. non-invasive. Non-deformed organoids were expressed relative to the 8 captured organoids for each experimental condition and plotted in bar graphs. Each experiments was repeated at least three independent times to allow statistical inference.

### Statistical analyses

All survival analyses were performed using Stata 12 (StataCorp LP). The event under study was DSOS, defined as time from diagnosis to death due to breast cancer. PIAS1 results were available for 96 patients. Whole tumor PIAS1 expression using AQUA was dichotomized by any vs no PIAS1 expression, and PIAS1 tumor nuclear to cytoplasmic ratio using HALO was dichotomized at its median. Kaplan-Meier curves were analyzed using the logrank test, and Cox proportional hazard regression was performed to estimate hazard ratios. Biochemical and organoid related data were subjected to statistical analysis by Student’s t-test or One-way ANOVA followed by Tukey-Kramer post-test using InStat (Graphpad InStat, San Diego, CA, USA). Values of P≤0.05 were considered statistically significant. *P≤0.05, **P≤0.01, and ***P≤0.001. Data were presented graphically as mean ± S.E.M. from experiments that were repeated at least three independent times.

## Results

### Protein abundance and nuclear localization of PIAS1 correlate with breast cancer patient survival

**Fig 1:**
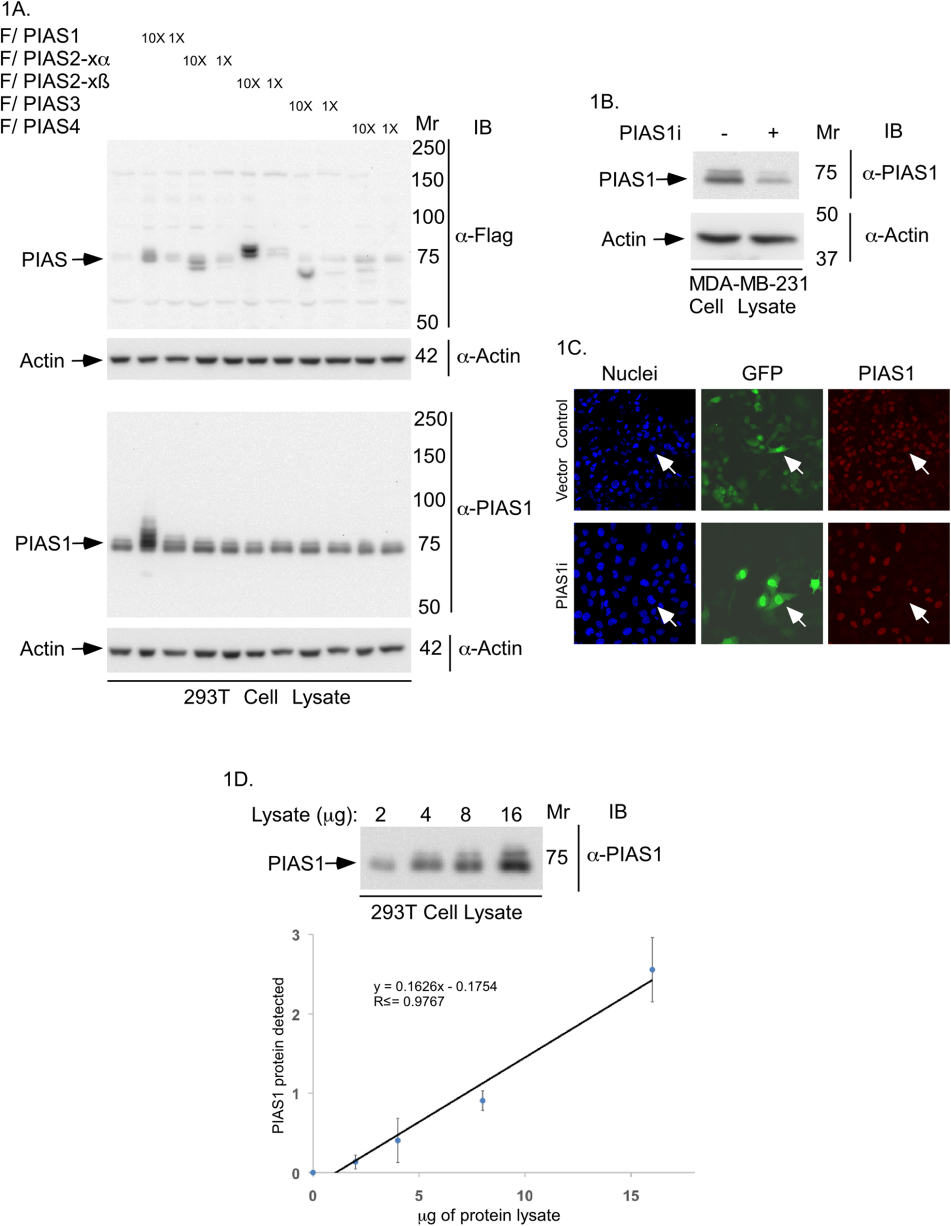
Characterization of the PIAS1 antibody. (A) PIAS1 and actin-immunoblots of lysate of 293T cells expressing one of two different concentrations (1X or 10X) of FLAG-tagged PIAS1, PIAS2-xα, PIAS2-xβ, PIAS3 or, PIAS4, or transfected with the vector control. Actin immunoblotting was used as loading control. Only PIAS1 antibody-immunoreactive bands corresponding to endogenous PIAS1 and exogenous FLAG tagged-PIAS1 were detected. Immunoblots are from an experiment that was repeated twice with similar results. (B) PIAS1 or actin immunoblots of lysate of MDA-MB-231 cells transiently transfected with an RNAi vector control or receiving a pool of plasmids expressing short hairpin RNAs against two distinct regions of PIAS1 [18,19]. Control and PIAS1 RNAi plasmids also express CMV-driven green fluorescence protein (GFP). Immunoblots are from an experiment that was repeated twice with similar results. (C) Representative PIAS1 (red), GFP (green) and nuclei (blue) fluorescence microscopy micrographs of MDA-MB-231 cells transfected as in B, and subjected to anti-PIAS1 indirect immunofluorescence and counterstained with Hoechst 33342 fluorescent nucleotide dye to visualize nuclei. GFP signal indicate control vector or PIAS1 RNAi plasmid-transfected cells. Arrow shows an example of each a vector transfected cell (upper row) and a PIAS1 RNAi transfected cell (lower row) to highlight the knockdown of endogenous PIAS1 in PIAS1i-transfected cell. These experiments were repeated two times with similar outcomes. (D) Representative PIAS1 immunoblots of serially-diluted lysate of 293T cells (upper panel), and protein abundance of PIAS1 quantified by densitometry (y-axis) plotted versus the protein amount of lysate (x-axis) (lower panel). The abundance of PIAS1 for each point in the XY graph is the mean ± SEM from three independent experiments including the one shown in upper panel. Regression analysis indicated that the protein abundance of PIAS1 follows a linear relationship with total protein amount in cells lysates.

The SUMO E3 ligase PIAS1 inhibits the invasive behaviour of breast cancer cells grown in a three-dimensional model system, and deregulation of PIAS1 activity promotes the growth of breast cancer cell-derived metastases in a xenograft mouse model [11]. These findings raise the key question of whether PIAS1 has prognostic value in breast cancer. We used tissue microarray (TMA) analysis to assess the protein abundance and localization of PIAS1 in human breast cancer. First, we thoroughly characterized the specificity of PIAS1 antibodies. We compared three commercially available PIAS1 antibodies. Based on initial immunoblotting findings, one of the three antibodies (Abcam Cat# ab109388) was characterized further. To evaluate the specificity of the PIAS1 antibody, we carried out immunoblotting analyses on lysates of 293T cells transfected with a vector control or expression plasmid encoding FLAG-tagged PIAS1, PIAS2-xα, PIAS2-xβ, PIAS3, or PIAS4 (Fig 1A). Immunoblotting with the PIAS1 antibody revealed that the Abcam PIAS1 antibody specifically recognized PIAS1 but no other PIAS family members (Fig 1A). We also performed immunoblotting of endogenous PIAS1 in MDA-MB-231 cells. A PIAS1 immunoreactive band of the appropriate size was reduced in MDA-MB-231 cells upon induction of PIAS1 knockdown by RNAi (Fig 1B), suggesting the Abcam antibody specifically recognizes endogenous PIAS1. PIAS1 is localized mainly within the nucleus [14,25]. Immunocytochemical analysis confirmed that the PIAS1 immunoreactivity was predominantly nuclear, which was reduced in PIAS1 knockdown cells (Fig 1C). Immunoblotting analyses of 293T cells, in which increasing amount of lysate was analyzed, confirmed the utility of the Abcam antibody in recognizing endogenous PIAS1 in a linear dynamic range (Fig 1D). Collectively, these data showed that the Abcam PIAS1 antibody is suitable for tissue microarray immunohistochemical analyses.

**Fig 2:**
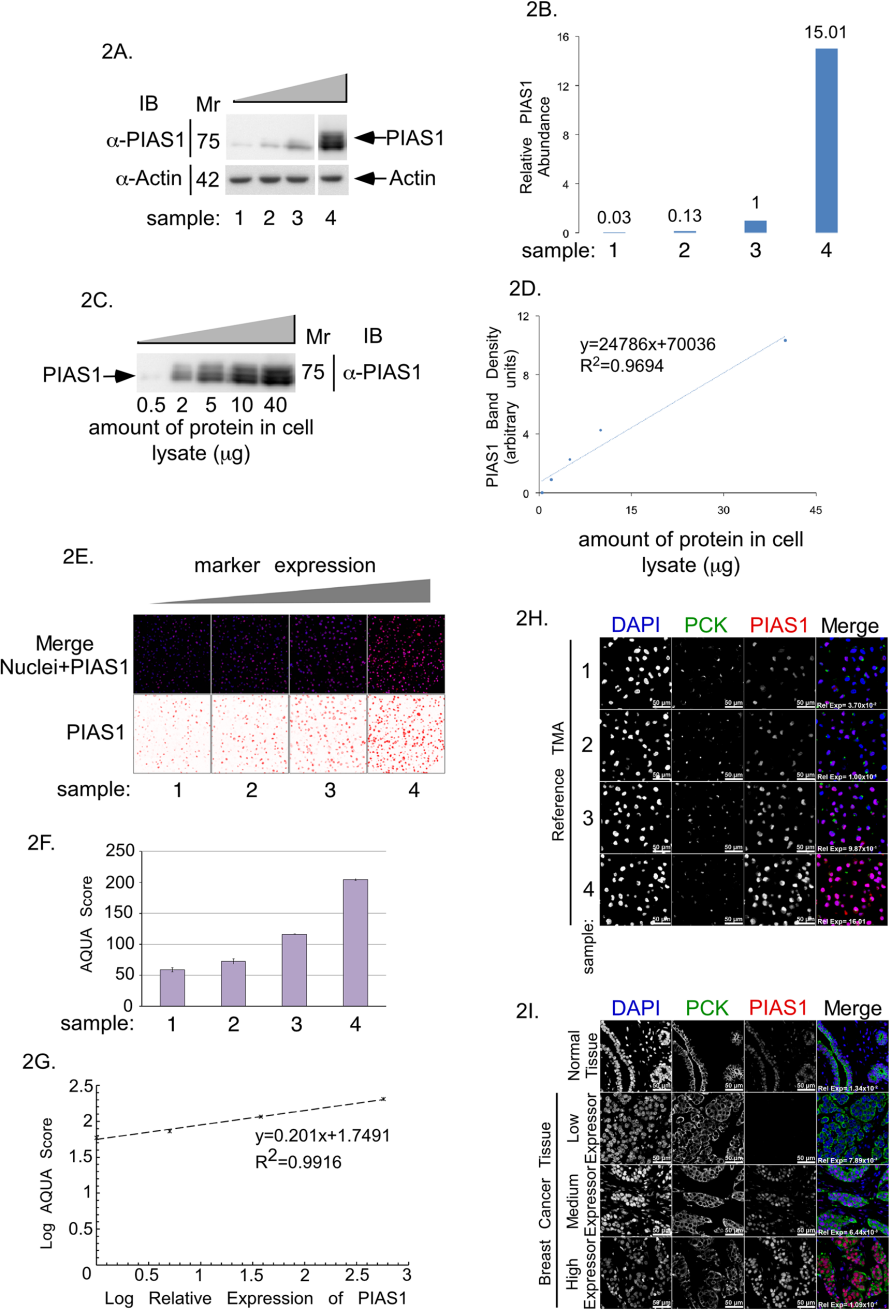
PIAS1 protein abundance analysis in reference TMA and breast cancer TMA. (A) PIAS1 and actin immunoblots of lysate of MDA-MB-231 cells expressing four increasing concentrations of PIAS1 (samples 1 to 4). (B) Bar graph of actin-normalized PIAS1 protein abundance in samples 1 to 4 shown in A and expressed relative to actin-normalized PIAS1 abundance in sample 3. (C) PIAS1 immunoblot of serially diluted lysate of MDA-MB-231 cells overexpressing PIAS1 in sample 4. (D) XY-graph plot of the lysate's total protein on the x-axis versus PIAS1 protein abundance, quantified from PIAS1immunoblot in C, on the y-axis. (E) Representative PIAS1 (red), and nuclei (blue) fluorescence micrographs of sections of Histogel-embedded MDA-MB-231 cells reference TMA expressing increasing abundance of PIAS1, corresponding to samples 1 to 4 in panel A, which were subjected to anti-PIAS1 indirect immunofluorescence and DAPI dye staining to visualize nuclei. (F) Bar graph depicts AQUA analysis software-quantified PIAS1 abundance in the reference TMA shown in E. (G) The XY-graph shows the relationship between Log of relative abundance of PIAS1 in samples 1 to 4 of MDA-MB-231 cell lysates quantified by immunoblotting on the x-axis versus Log of abundance of PIAS1 in MDA-MB-231 samples 1 to 4 of reference TMA quantified by AQUA analyses of immunocytochemistry on the y-axis. (H) Representative fluorescence microscopy micrographs of histogel-MDA-MB-231 cell reference TMA blocks. (I) Representative fluorescence microscopy micrographs of paraffin-embedded normal breast tissue and examples of three breast cancer tissues expressing different amounts of PIAS1. For both H and I, TMA were subjected to anti-PIAS1 (red) and anti-Pan cytokeratin (green) antibodies indirect immunofluorescence, and nuclear counterstaining with DAPI (blue). PIAS1-Cytokeratin-Nuclei merged fluorescence micrograph panels show relative abundance of PIAS1 in reference breast cancer TMA, normal breast and breast cancer tissue array.

Immunohistochemistry (IHC) is a cost-effective and versatile method for detecting, visualizing and quantifying specific proteins in tissue samples, and is thus commonly used as a biomarker discovery tool. However, the validity of such studies is significantly hampered by poor reproducibility, in part due to lack of reference materials. To ensure appropriate range of detection, and to further characterize the reproducibility of the PIAS1 biomarker assay in this study, we established reference materials for assay calibration. Reference material comprised human breast cancer MDA-MB-231 cells where the abundance of endogenous PIAS1 was decreased by PIAS1 RNAi knockdown (samples 1 and 2), left unchanged (sample 3), or increased by overexpression (sample 4) to provide increasing cellular concentrations of PIAS1 (Fig 2A and 2B). Immunoblotting of serially diluted lysate of the cells with the highest PIAS1 expression (Fig 2A, sample 4) was used to generate a standard curve (Fig 2C and 2D), which facilitated the measurement of the relative abundance of PIAS1 in MDA-MB-231 cells (Fig 2A and 2B). To generate a reference TMA, MDA-MB-231 cells expressing increasing protein concentrations of PIAS1 (Fig 2A) were embedded within commercial Histogel and subjected to immunocytochemical analyses using the PIAS1 antibody (Fig 2E), followed by semi-quantitative image evaluations using the Genoptix Automated Quantitative Analysis (AQUA) algorithm (Fig 2F). Importantly, we found that the PIAS1-immunofluorescence AQUA scores from the MDA-MB-231 cell-derived reference TMA correlated linearly with the relative protein abundance of PIAS1 measured by immunoblotting of corresponding lysates (Fig 2G). In addition, relative expression of PIAS1 obtained by Genoptix AQUA and Indica Lab HALO analysis platforms followed a linear relationship (S1 Fig).

**Fig 3:**
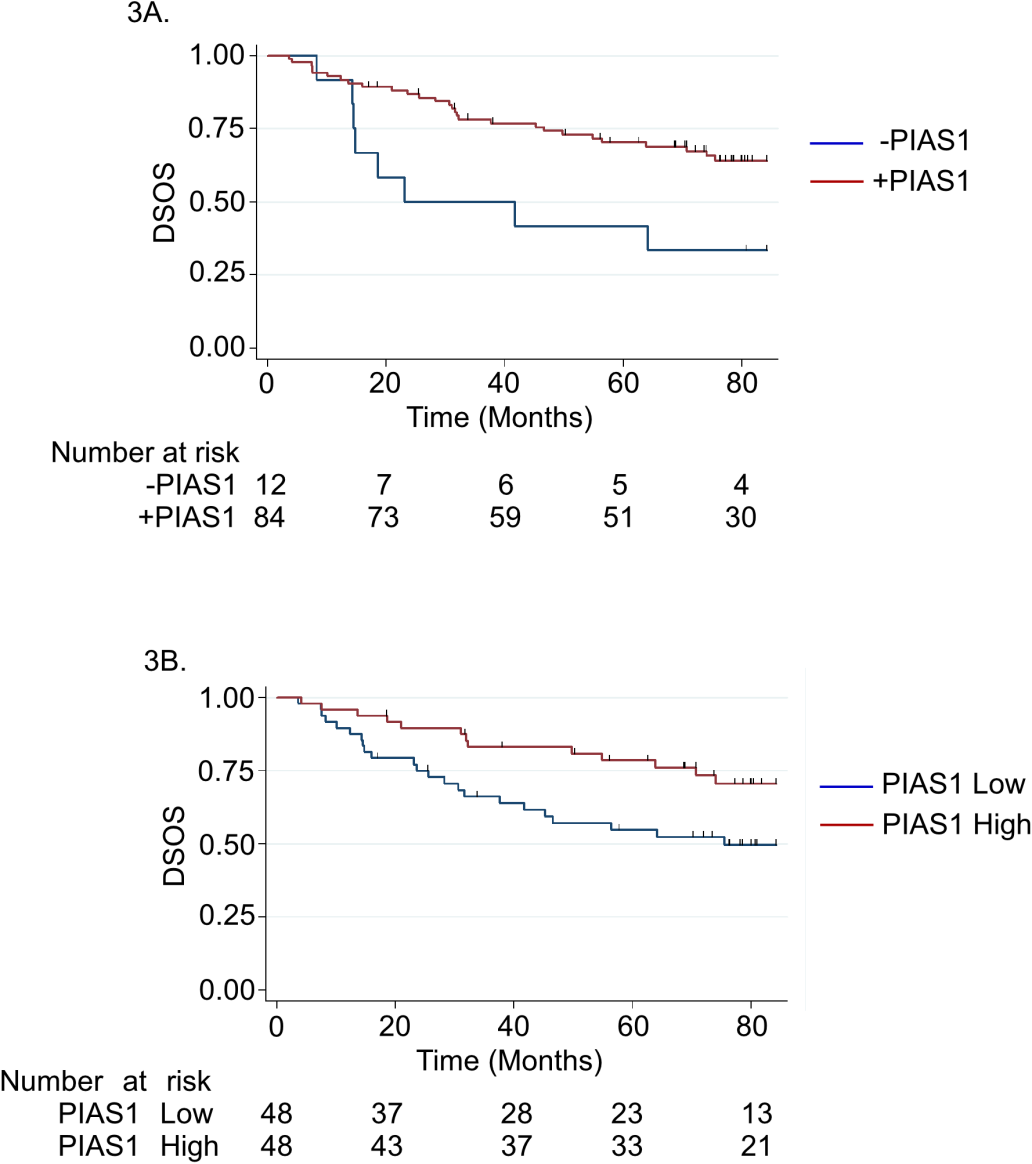
PIAS1 as a potential positive predictor of overall survival in breast cancer. (A) Kaplan Meier survival curves showing univariate analysis with number of individuals at risk for each group detailed in table below for whole tumor PIAS1 expression by AQUA, no expression vs any expression (logrank p-value = 0.0127) suggest that patients with any PIAS1 tumor expression were less likely to have a DSOS event. (B) Kaplan Meier survival curves showing univariate analysis with number at risk for each group detailed in table below for PIAS1 tumor nuclear expression relative to PIAS1 tumor cytoplasmic expression by HALO (logrank p-value = 0.0246) suggest that patients who had higher PIAS1 tumor nuclei expression, relative to the tumor cytoplasm, had a more favorable DSOS. Number at risk refers to the number of patients who are still alive in each group at the specified time point as indicated by the x-axis of the Kaplan Meier graph.

Using the reference array, we then analyzed the relative abundance and subcellular localization of PIAS1 in a subset of patients from the Calgary tamoxifen cohort [20]. This cohort comprises a single TMA consisting of 130 patients (65 patients with no recurrence vs 65 patients who had a recurrence, both assessed within 5 years of initial diagnosis, hereon referred to as the Calgary 65/65 cohort) [21]. Of the 130 patients in the Calgary-65/65 cohort, we assessed TMAs and outcome data for 108 patients who met the inclusion/exclusion criteria (see Materials and Methods). We also evaluated the clinical-pathological details and their relation to disease-specific overall survival (DSOS) (Table 1). Among the 108 patients, 96 had sufficient tissue that could be evaluated. Using the XY-standard curve (Fig 2G), the relative abundance of PIAS1 in the reference and actual TMAs was determined by linear interpolation/extrapolation method (Fig 2H and 2I). Using AQUA analyses, we found that patients with any detectable amount of PIAS1 were less likely to have a DSOS event (death due to breast cancer) [HR = 0.38, 95% confidence interval (CI): 0.17–0.84, p = 0.016] (Fig 3A). We also characterized the protein abundance of PIAS1 using HALO. In these analyses, patients who had higher amount of PIAS1 in tumor nuclei, relative to the tumor cytoplasm (dichotomized at median), had a more favourable DSOS [HR = 0.47, 95% CI: 0.24–0.92, p = 0.028) (Fig 3B). Thus, AQUA and HALO-based analyses suggest that the protein abundance and subcellular localization of PIAS1 in the TMA correlated with breast cancer patient DSOS. Moreover, the relationship between the abundance of PIAS1 or its subcellular localization to DSOS held up even in a multivariate model, adjusting for tumor size and lymph node status (HR = 0.46, (95%CI: 0.22–0.95); p = 0.035). Together, these findings suggest that the protein abundance and nuclear localization of PIAS1 predict improved survival in breast cancer patients.

**Table 1:** Correlation of clinicopathological characteristics and DSOS in Calgary 65/65 cohort.

	N = 108		Disease Specific Overall Survival			
Variable	n	%	No Event	Event	p-value	TEST
**AGE**						
Median	69.9		69	39	0.004	Wilcoxon
Range	37.9–86.7					
< 53	17	15.7	16	1	0.005	Fisher's Exact
≥ 53	91	84.3	53	38		
**TUMOUR SIZE (cm)**						
Median	2.0		68	37	0.0002	Wilcoxon
Range	0.4–11.0					
< 2	48	44.4	40	8	<0.001	Chi2
≥ 2	57	52.8	28	29		
Missing	3	2.8				
**GRADE**						
1	20	18.5				
2	54	50.0				
3	27	25.0				
Unknown	7	6.5				
Low (1/2)	74	68.5	53	21	0.012	Chi2
High (3)	27	25.0	12	15		
Missing	7	6.5				
**NODE STATUS**						
Negative	55	50.9	47	8	<0.001	Chi2
Positive	38	35.2	13	25		
Missing	15	13.9				
**ER Status**						
Negative	10	9.3	6	4	1.000	Fisher's Exact
Positive	98	90.7	63	35		
**PR Status**						
Negative	23	21.3	14	9	1.000	Fisher's Exact
Positive	85	78.7	55	30		
**HER2 Status**						
Negative	103	95.4	66	37	1.000	Fisher's Exact
Positive	5	4.6	3	2		
**Whole Tumor PIAS1**						
Negative	12	11.1	4	8	0.052	Fisher's Exact
Positive	84	77.8	56	28		
Missing	12	11.1				
**PIAS1 Tumor Nuclear:Cytoplasm**						
Negative	48	44.4	25	23	0.035	Chi2
Positive	48	44.4	35	13		
Missing	12	11.1				

### PIAS1 regulates the invasive growth of breast cancer cell-derived organoids via SnoN sumoylation

The findings that the protein abundance or nuclear localization of PIAS1 correlates with breast cancer patient survival led us next to determine the mechanism by which PIAS1 might regulate the malignant behaviour of breast cancer cells. Notably, PIAS1 acts in a SUMO E3 ligase-dependent manner to supress the invasive and metastatic growth of breast cancer cells [11]. In non-transformed epithelial cells, we have also found that PIAS1 suppresses EMT via sumoylation of the transcriptional regulator SnoN [12]. These observations raised the question of whether PIAS1 might regulate the malignant behaviour of breast cancer cells via sumoylation of SnoN.

**Fig 4:**
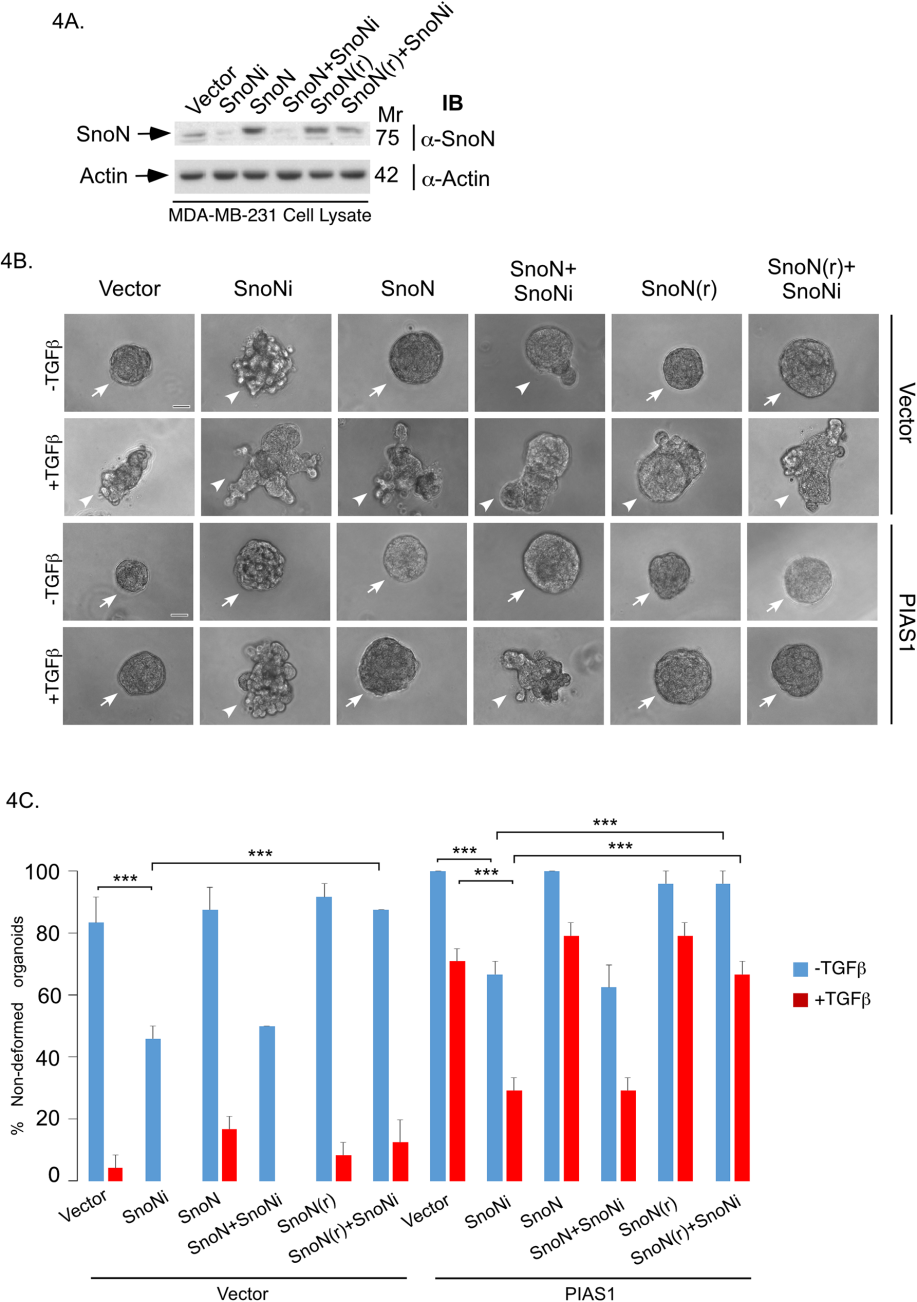
Endogenous SnoN mediates the ability of PIAS1 to suppress the invasive growth of breast cancer cell-derived organoids. (A) SnoN and actin immunoblots of lysate of MDA-MB-231 cells cotransfected with a plasmid encoding a short hairpin RNA targeting a specific region of SnoN mRNA or a control plasmid, together with an expression plasmid encoding an RNAi-resistant SnoN (SnoN(r)) or wild type RNAi-sensitive SnoN, or the corresponding vector control. (B) Representative DIC light microscopy micrographs of untreated or 100pM TGFβ-treated 8-day old three-dimensional organoids derived from MDA-MB-231 cells stably expressing PIAS1 or the corresponding vector control, and co-transfected with SnoN RNAi, SnoN(r), wild type SnoN, or respective control plasmids. (C) Bar graph depicts mean ± SEM proportion of non-deformed organoids expressed as a percentage of total colonies counted for each experimental condition from three independent experiments including the one shown in B. Non-deformed organoids represents non-invasive growth phenotype. Knockdown of endogenous SnoN promoted invasive growth of organoids as compared to vector control or PIAS1 stably expressing cells in the absence or presence of TGFβ. Expression of SnoN(r) but not wild type SnoN suppressed the ability of SnoN RNAi to promote invasive growth of organoid in vector control or PIAS1 expressing cells in the absence or presence of TGFβ. Significant difference, ANOVA: ***P≤0.001. Scale bar indicates 50 μm. Arrows and arrowheads indicate non-deformed and invasive organoids, respectively.

We used a three-dimensional breast cancer cell-derived organoid model to determine the role of SnoN and its sumoylation in breast cancer cell invasiveness [11,26]. We employed the human triple negative MDA-MB-231 breast cancer cells in a three-dimensional culture system, where single MDA-MB-231 cells were grown in Matrigel, which provides extracellular matrix support to better reflect *in vivo* setting. Three-dimensional MDA-MB-231 cell-derived organoids were monitored, and images captured at day 8 of culture. MDA-MB-231 cells formed filled multicellular spherical colonies with some protrusions (Fig 4B, [11]). Non-transformed mammary epithelial cells usually organize into hollow acini when grown in three-dimensional cultures [10,24]. The filled phenotype of MDA-MB-231 cell-derived organoids reflects their transformed nature as evidenced by their EMT-like characteristics in conventional two-dimensional cultures [11,27]. Three-dimensional MDA-MB-231 cell-derived organoids undergo deformation and invasive behaviour, characterized by extensive budding, protrusions, and disorganization of these organoids upon exposure to the cytokine TGFβ (Fig 4B, [11]).

We determined the effect of RNAi-mediated knockdown of endogenous SnoN (Fig 4A) on three-dimensional MDA-MB-231 cell-derived organoids in the absence or presence of TGFβ. Knockdown of endogenous SnoN promoted the invasive growth of MDA-MB-231 cell-derived organoids as indicated by significant reduction in the percent spherical organoids even in the absence of TGFβ addition (Fig 4B and 4C). Coexpression of an RNAi-resistant form of SnoN (SnoN (r)) suppressed the SnoN RNAi-induced invasive behaviour of three-dimensional MDA-MB-231 cell-derived organoids, excluding the possibility that the SnoN RNAi-effect is due to off target effect (Fig 4A, 4B and 4C). These data suggest that endogenous SnoN maintains non-invasive multicellular structures of the three-dimensional MDA-MB-231 cell-derived organoids.

We also determined the role of endogenous SnoN in the ability of overexpressed PIAS1 to suppress TGFβ-induced invasive behaviour of MDA-MB-231 cell-derived organoids (Fig 4B and 4C). Remarkably, endogenous SnoN knockdown reduced the ability PIAS1 to suppress the invasive growth of MDA-MB-231 cell-derived organoids in the absence or presence of TGFβ (Fig 4B and 4C). In addition, SnoN(r), but not the RNAi-sensitive SnoN, reversed the ability of SnoN RNAi to suppress the PIAS1-induced phenotype in three-dimensional MDA-MB-231 cell cultures (Fig 4B and 4C). These findings suggest that endogenous SnoN mediates the ability of PIAS1 to suppress TGFβ-induced invasive growth of breast cancer cells.

**Fig 5:**
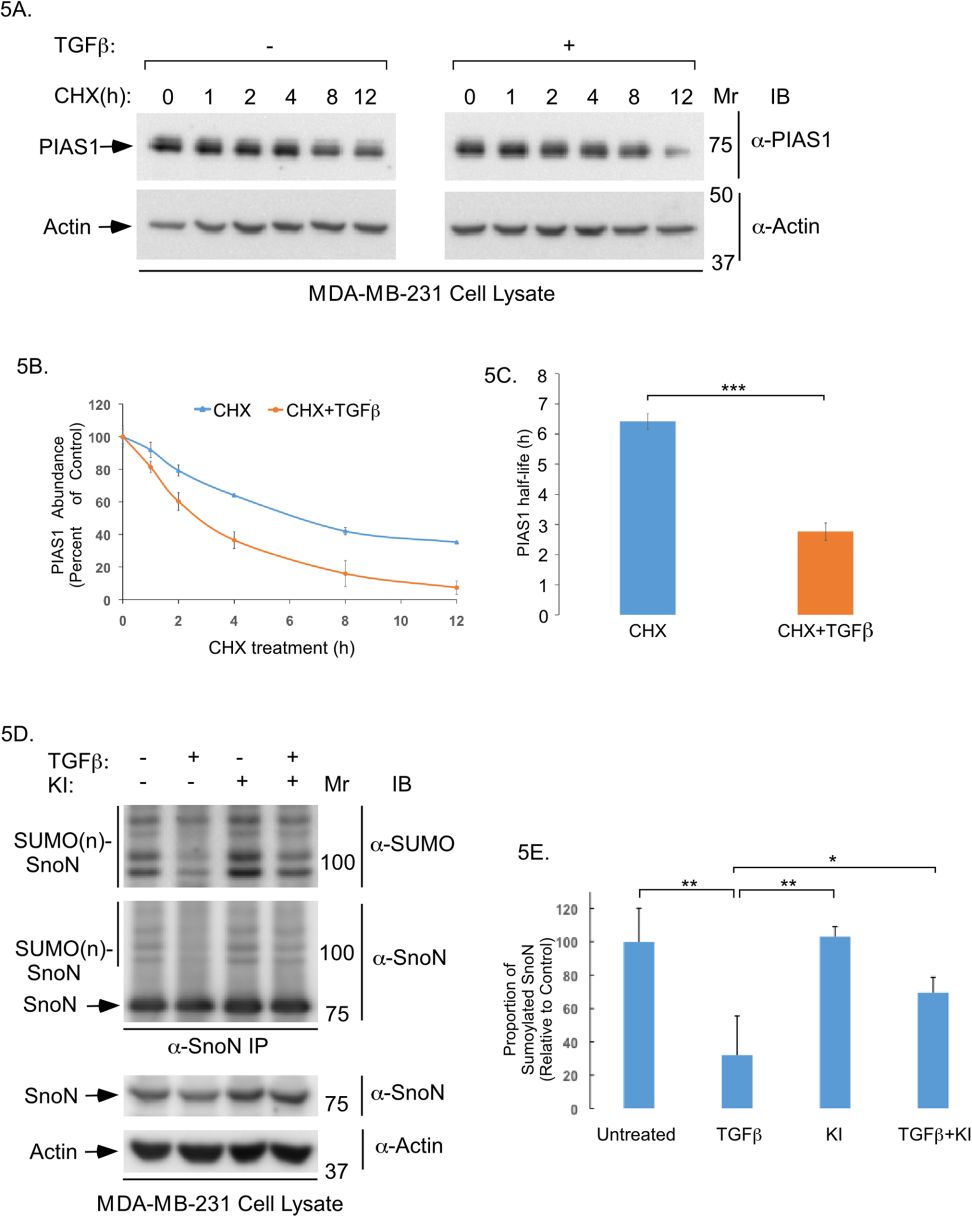
TGFβ regulates PIAS1 protein stability and SnoN sumoylation abundance in breast cancer cells. (A) PIAS1 and actin immunoblots of lysates of MDA-MB-231 cells pre-incubated without or with 100pM TGFβ for 12 hours prior to treatment with 10μg/ml cycloheximide (CHX) for 0, 1, 2, 4, 8 and 12 hours (h). (B) An XY-graph of CHX treatment (h) on x-axis versus mean ± SEM (n = 3 experiments including one shown in A) of relative actin-normalized PIAS1 abundance in cells pre-incubated without (blue line) or with TGFβ (red line) on the y-axis. TGFβ increases the PIAS1 protein abundance turnover. (C) Bar graph represents the mean ± SEM of PIAS1 protein half-life in MDA-MB-231 cells pre-incubated without (blue) or with TGFβ (red) quantified from three-independent experiments including from the experiment shown in A. Half-life of PIAS1 protein was determined from graph shown in B by interpolation. TGFβ reduced PIAS1 protein half-life by 57%. (D) SUMO1 and SnoN immunoblots (panels 1 and 2) of NEM-treated SnoN immunoprecipitation (SnoN IP) of lysate of SnoN-expressing MDA-MB-231 cells incubated for 12 hours without or with TGFβ, KI, alone or together. Unmodified SnoN has a MW of 77 KDa (arrow), and sumoylated SnoN protein species run as 100 KDa and higher as detected in SUMO and SnoN immunoblots of SnoN immunoprecipitation (SUMO(n)SnoN, vertical lines) [12,18]. SnoN and actin immunoblots (panels 3 and 4) of lysates of cells treated as described above for panels 1 and 2 are also shown. (E) The bar graph represents the mean ± SEM of proportion of sumoylated SnoN relative to unmodified SnoN quantified from SnoN immunoblots of SnoN immunoprecipitation and expressed relative to the proportion of sumoylated SnoN in the untreated control from three independent experiments including the one shown in D. TGFβ-signalling reduces the proportion of sumoylated SnoN by 68% in MDA-MB-231 cells. Statistical difference, (C) Student t-test: ***P≤ 0.001, (E) ANOVA: **P≤0.01, *P≤0.05.

TGFβ promotes the ubiquitin-mediated degradation of PIAS1 and downregulates sumoylation of SnoN in non-transformed epithelial cells [12]. The findings that SnoN suppresses TGFβ-induced invasive growth of MDA-MB-231 cell-derived organoids in the presence of PIAS1 (Fig 4B and 4C) led to prediction that PIAS1 abundance and SnoN sumoylation might also be regulated by TGFβ signalling in MDA-MB-231 cells. Cycloheximide-based analyses showed that TGFβ stimulation significantly reduced the half-life of endogenous PIAS1 in MDA-MB-231 cells (Fig 5A, 5B and 5C). TGFβ signalling also reduced the proportion of sumoylated SnoN in MDA-MB-231 cells (Fig 5D and 5E).

**Fig 6:**
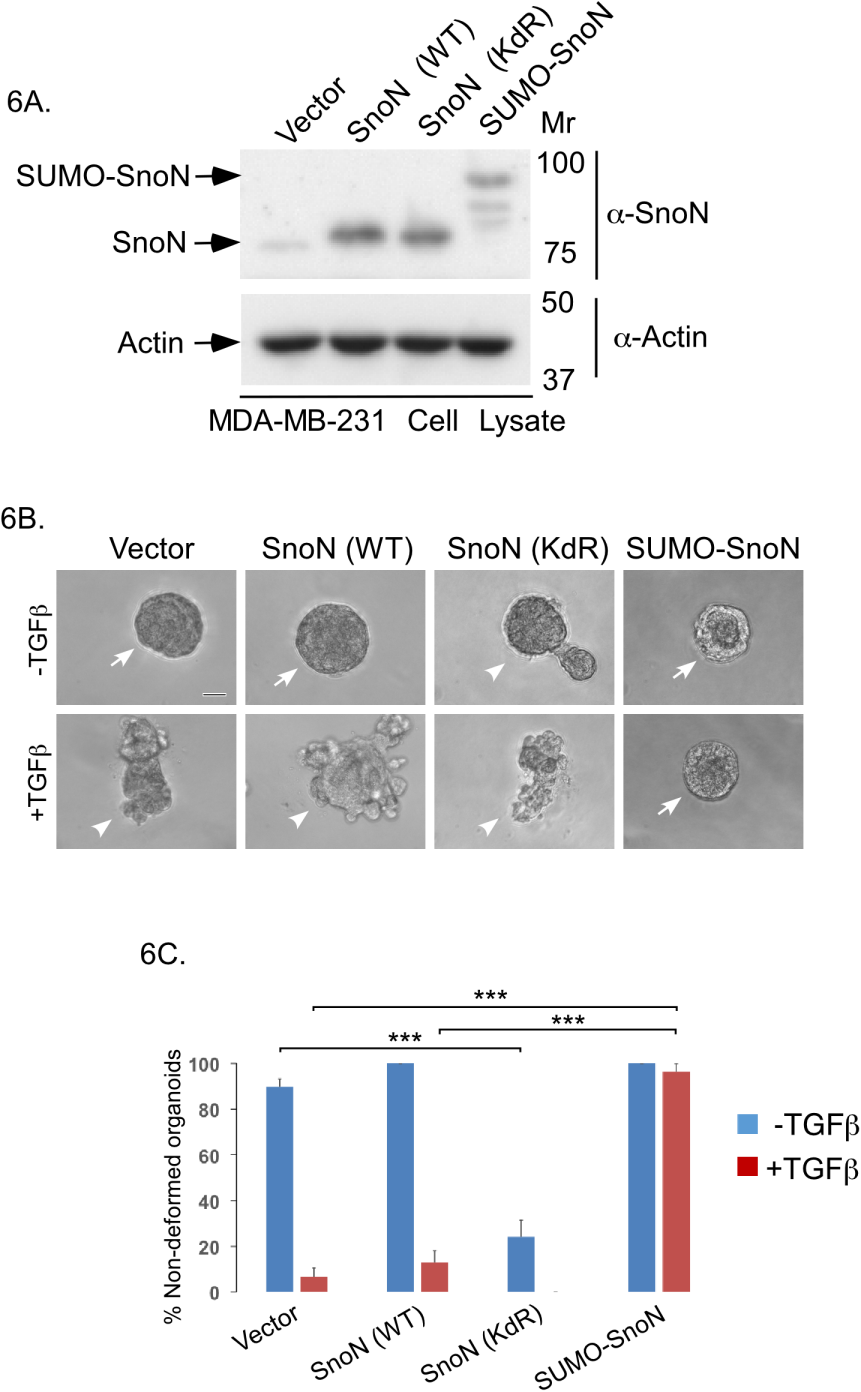
Sumoylation of SnoN supresses TGFβ-induced invasiveness of breast cancer organoids. (A) SnoN and actin immunoblots of lysates of MDA-MB-231 cells expressing SnoN (WT), SnoN (KdR), SUMO-SnoN or transfected with a control vector. (B) Representative DIC light microscopy micrographs of untreated or 100pM TGFβ-treated 8-day old three dimensional organoids derived from MDA-MB-231 cells transfected as in A. (C) Bar graph represents mean ± SEM proportion of non-deformed organoids expressed as a percentage of total colonies counted for each experimental condition from four independent experiments including the one shown in B. Non-deformed organoids represents non-invasive growth phenotype. SnoN (KdR) promoted an invasive growth of breast cancer cell-derived organoids even in the absence of TGFβ. SUMO-SnoN suppressed TGFβ-induced invasive growth of breast cancer cell-derived organoids. Significant difference, ANOVA: ***p<0.001. Scale bar indicates 50 μm. Arrows and arrowheads indicate non-deformed and invasive organoids, respectively.

PIAS1 triggers the sumoylation of SnoN at Lysines 50 and 383 in epithelial cells [12]. Therefore, we asked whether PIAS1 suppresses the invasive behaviour of breast cancer cells via SnoN sumoylation. We first compared the ability of wild type SnoN (SnoN (WT)), a SUMO loss of function SnoN, in which Lysines 50 and 383 are converted to arginine (SnoN (KdR)), and a SUMO gain of function SnoN, in which a SENP-insensitive SUMO protein is fused to SnoN (SUMO-SnoN), whose expression were confirmed by immunoblotting (Fig 6A), on three-dimensional MDA-MB-231 cell-derived organoids in the absence or presence of TGFβ [12,18,24]. We found that SUMO-SnoN suppressed the ability of TGFβ to induce invasive growth of the breast cancer cell-derived organoids. In contrast, SnoN (KdR) induced the invasive growth of three-dimensional MDA-MB-231 cell-derived multicellular structures even in the absence of addition of TGFβ (Fig 6B and 6C).

**Fig 7:**
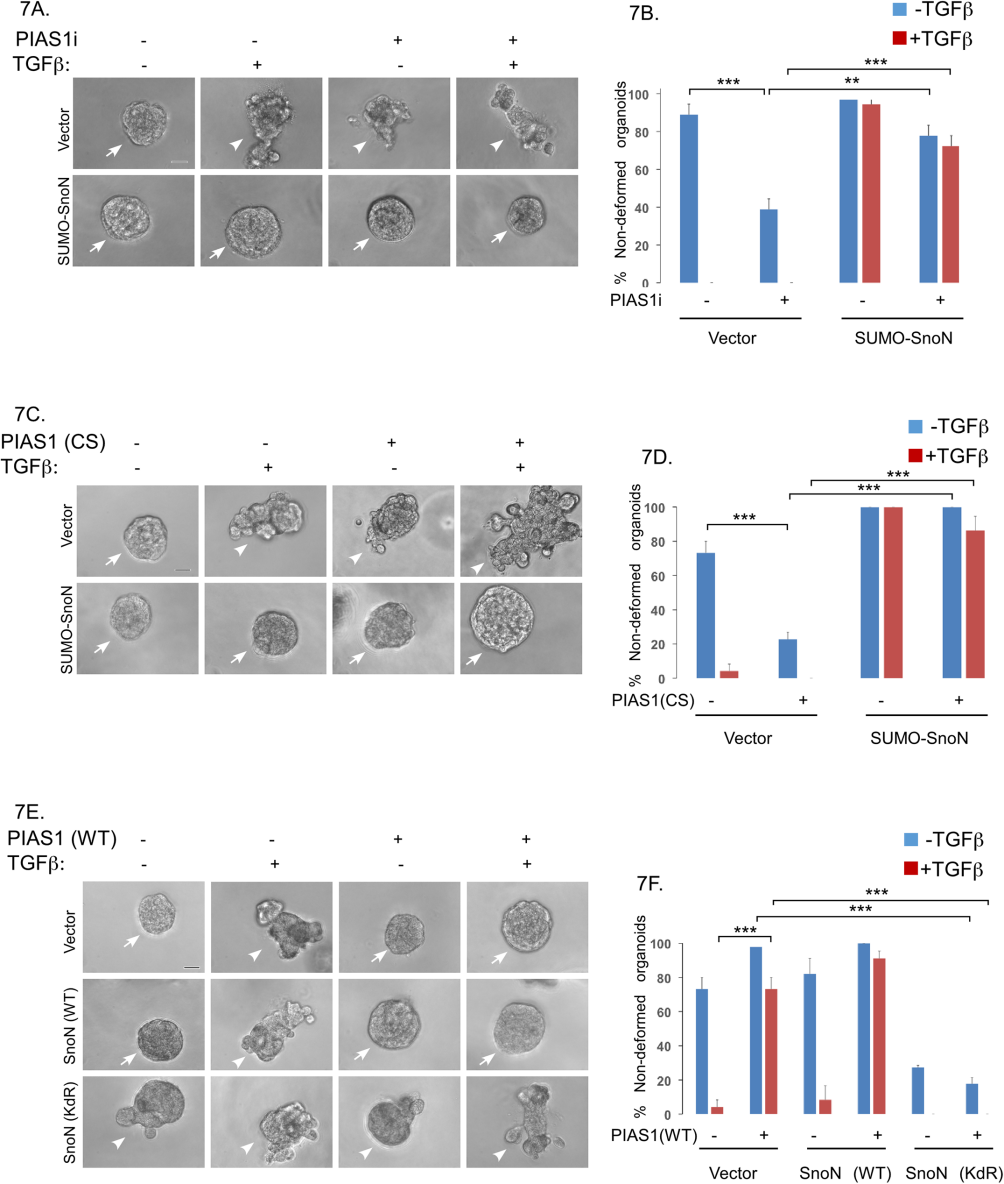
PIAS1 suppresses TGFβ-induced budding and disruption of breast cancer cell-derived organoids via sumoylation of SnoN. (A) Representative DIC light microscopy micrographs of untreated or 100pM TGFβ-treated 8-day old organoids derived from MDA-MB-231 cells stably expressing SUMO-SnoN or the control vector, and transfected with a pool of plasmids encoding short hairpin RNAs targeting distinct regions of PIAS1 mRNA, or a control RNAi plasmid. (B) Bar graph represents mean ± SEM proportion of non-deformed organoids expressed as a percentage of total colonies counted for each experimental condition from three independent experiments including the one shown in A. SUMO-SnoN suppressed the ability of PIAS1 knockdown to promote invasive growth of MDA-MB-231 cell-derived organoids in absence or presence of TGFβ. (C) Representative DIC light microscopy micrographs of untreated or 100pM TGFβ-treated 8-day old organoids derived from MDA-MB-231 cells stably expressing SUMO-SnoN or the control plasmid, and transfected with a vector expressing PIAS1 (CS) or the control vector. (D) Bar graph represents mean ± SEM proportion of non-deformed organoids expressed as a percentage of total colonies counted for each experimental condition from three independent experiments including the one shown in C. SUMO-SnoN suppressed PIAS1 (CS)-induction of invasive growth of MDA-MB-231 cell-derived organoids in absence or presence of TGFβ. (E) Representative DIC light microscopy micrographs of untreated or 100pM TGFβ-treated 8-day old organoids derived from MDA-MB-231 cells stably expressing SnoN (WT), SnoN (KdR) or a vector control, and transfected with PIAS1 (WT) or the control vector. (F) Bar graph depicts mean ± SEM proportion of non-deformed organoids expressed as a percentage of total colonies counted for each experimental condition from three independent experiments including the one shown in E. SnoN (KdR) antagonized the ability of PIAS1 (WT) to suppress the invasive growth of MDA-MB-231 cell-derived organoids in absence or presence of TGFβ. Significant difference, ANOVA: ***P≤0.001, **P≤0.01. Scale bar indicates 50 μm. Non-deformed organoids represents non-invasive growth phenotype. Arrows and arrowheads indicate intact non-deformed and invasive organoids, respectively.

Next, we assessed the role of sumoylated SnoN in the ability of endogenous PIAS1 to maintain the non-invasive behaviour of MDA-MB-231 cell-derived organoids. As expected, knockdown of PIAS1 led to disruption and deformation of MDA-MB-231 cell-derived organoids even in the absence of TGFβ [11]. However, we found that SUMO-SnoN suppressed the ability of PIAS1 knockdown to induce the invasive growth of MDA-MB-231 cell-derived organoids (Fig 7A and 7B). In other experiments, we tested the effect of SnoN sumoylation on the ability of a SUMO E3 ligase defective PIAS1, in which Cysteine 350 is converted to serine (PIAS1 (CS)), to promote the invasive behaviour of MDA-MB-231 cell-derived organoids. PIAS1 (CS) phenocopied PIAS1-knockdown in promoting disorganization and deformation of the MDA-MB-231-derived organoids even in the absence of TGFβ (Fig 7C and 7D). Importantly, coexpression of SUMO-SnoN reversed the ability of PIAS1 (CS) to disrupt MDA-MB-231 cell-organoids (Fig 7C and 7D). Conversely, coexpression of SnoN (KdR) antagonized the ability of overexpressed PIAS1 to suppress TGFβ-induced invasive growth of three-dimensional breast cancer cell-derived organoids (Fig 7E and 7F). Collectively, these data support the idea that PIAS1 acts via sumoylation of SnoN to suppress the invasive growth of breast cancer cells.

In summary, our findings have revealed an important role for PIAS1 as a predictive biomarker for breast cancer patient outcome. In addition, our findings suggest that PIAS1-SnoN sumoylation pathway may play a crucial role in suppression of the invasive growth of breast cancer cell-derived organoids.

## Discussion

In this study, we have identified the SUMO E3 ligase PIAS1 as a biologically relevant biomarker in breast cancer that predicts patient survival. We have found that the abundance of PIAS1 and its nuclear localization correlate positively with disease specific overall survival of breast cancer patients. In mechanistic studies, we have found that PIAS1 suppresses the invasive behaviour of human breast cancer cell-derived organoids via sumoylation of the transcriptional regulator SnoN. These findings define potentially crucial roles for PIAS1 in breast cancer pathogenesis, with significant implications for our understanding of breast cancer biology and management.

The identification of PIAS1 abundance and nuclear localization as potential useful biomarkers in prognosis of breast cancer is significant, in view of the scarcity of useful biomarkers in this disease. Consistent with our findings, the protein abundance and nuclear localization of PIAS1 has been suggested to correlate with patient survival [28]. PIAS1 has also been suggested to promote breast cancer cell apoptosis [29]. Interestingly, in a PIAS1 TMA-based study of primary breast tumors, where correlation with patient survival data were not reported, PIAS1 was found to be increased in abundance in breast tumor-derived tissue [30]. However, in this TMA study, PIAS1 was localized to the cytoplasm [30]. Our findings suggest that nuclear localization of PIAS1 predicts improved breast cancer patient survival. However, the significance of cytoplasmic PIAS1 localization as a prognostic biomarker remains to be clarified. Cytoplasmic localization of PIAS1 might inhibit its nuclear SUMO E3 ligase activity towards specific substrates such as SnoN, which is nuclear [28,31–33]. In the current study, we employed rigorous approaches to validate the specificity, selectivity and linear dynamic range of the PIAS1 antibody employed in the tissue microarray analysis. We also used two commercially available analysis platforms AQUA and HALO to analyze immunohistochemical data. Consistent with our findings PIAS1 has been suggested as a useful prognostic biomarker in other epithelial tumors including colon and gastric tumors [34,35]. On the other hand, PIAS1 has been suggested to predict poor outcome in patients with prostate cancer [36]. Thus, PIAS1 may serve as a general biomarker in cancer with positive or negative relationship to survival dependent on cancer types.

The potential utility of PIAS1 as a biomarker in predicting breast cancer patient survival is buttressed by new insights into the role of PIAS1 in breast cancer pathogenesis. PIAS1 suppresses the malignant behaviour human breast cancer cells in organoid cultures [11]. Our study advances our understanding of the mechanisms by which PIAS1 suppresses the invasive behaviour of breast cancer cell-derived organoids. PIAS1 triggers sumoylation of the transcriptional regulator SnoN, which mediates the ability of PIAS1 to suppress the invasive growth of three-dimensional breast cancer cell-derived organoids. These findings suggest that sumoylated SnoN might also serve as a biomarker in breast cancer. Interestingly, the abundance and nuclear localization of SnoN has been shown to be associated with lower grade tumors and predicts favourable outcome in breast cancer patients [37,38]. These observations suggest sumoylation might represent a novel mechanism by which SnoN may suppress cancer progression. Notably, in addition to PIAS1, recent studies have uncovered a novel role for TIF1γ as a SUMO E3 ligase for SnoN [24]. TIF1γ has been associated positively or negatively with different types of cancer including breast [39–41]. In future studies, it will be interesting to determine whether TIF1γ-induced SnoN sumoylation also regulates tumor invasiveness and metastasis, and TIF1γ acts as a potential biomarker.

In addition to SnoN, PIAS1 triggers sumoylation of other proteins including c-myc [42], Mdm2 [31] and AIB1 [43]. Thus, it would be interesting to evaluate the contribution of other PIAS1 SUMO substrates in its potential ability to regulate breast cancer invasiveness and metastasis, and to its utility as a biomarker in breast cancer.

In conclusion, we have demonstrated the prognostic value of PIAS1 in breast cancer and elucidated a mechanism for its potential action in the malignant behaviour of breast cancer. Our study thus advances our knowledge about the potential regulators of breast cancer invasion and metastasis. Based on the results of this study, future work on establishment of PIAS1-SnoN sumoylation axis as a therapeutic target in the clinical setting should be investigated. Studies on screening for drugs that stabilize PIAS1 in breast cancer should be performed to determine effect on cancer cell invasion and metastasis. Thus, our study opens up a potentially new avenue for breast cancer treatment and management.

## Supporting information

S1 Fig Comparison of two image-analysis platforms. The breast cancer TMA stained for the protein of interest was analyzed using HALO and AQUA platforms. 65/65 TMA's total cellular abundance of PIAS1 obtained by AQUA correlated linearly with that obtained by HALO (linear regression R^2^ = 0.8769, Spearman correlation = 0.9539).
(TIF)
