# Supplementary figures and images for: Identification of the SUMO E3 ligase PIAS1 as a potential survival biomarker in breast cancer
Source: PLoS One. 2017 May 11;12(5):e0177639. doi: 10.1371/journal.pone.0177639 (PMC5426774; doi:10.1371/journal.pone.0177639)

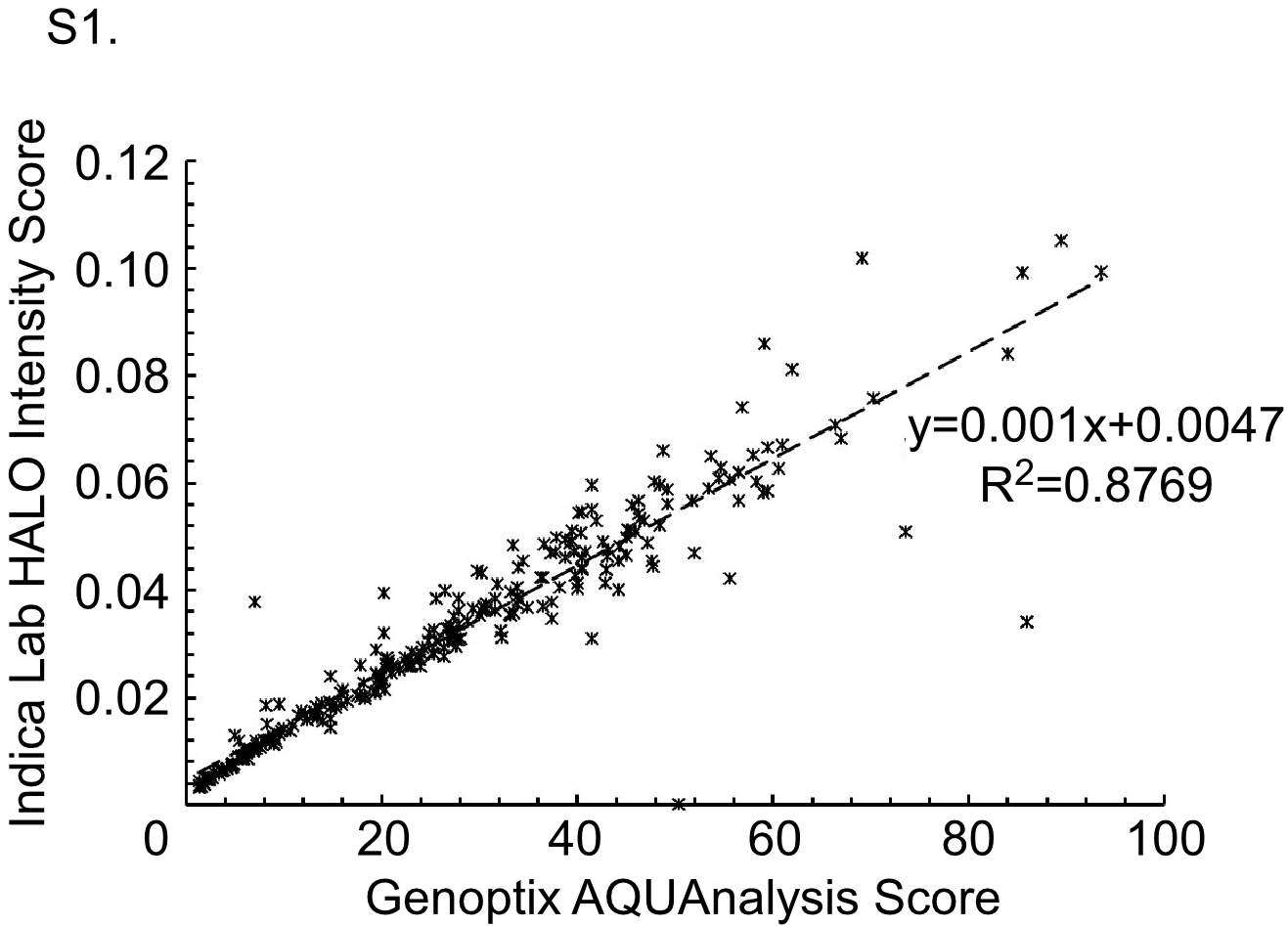

Supplement: S1 Fig — The breast cancer TMA stained for the protein of interest was analyzed using HALO and AQUA platforms. 65/65 TMA's total cellular abundance of PIAS1 obtained by AQUA correlated linearly with that obtained by HALO (linear regression R2 = 0.8769, Spearman correlation = 0.9539). (TIF) [file pone.0177639.s001.tif]
